# Analysing factors underlying the reporting of established non-native species

**DOI:** 10.1038/s41598-025-96133-0

**Published:** 2025-04-10

**Authors:** Phillip J. Haubrock, Ismael Soto, Ross N. Cuthbert, Irmak Kurtul, Elizabeta Briski

**Affiliations:** 1 Department of River Ecology and Conservation, Senckenberg Research Institute and Natural History Museum Frankfurt, Gelnhausen, Germany; 2 Faculty of Fisheries and Protection of Waters, University of South Bohemia in České Budějovice, South Bohemian Research Centre of Aquaculture and Biodiversity of Hydrocenoses, Zátiší 728/II, Vodňany, 389 25 Czech Republic; 3 Center for Applied Mathematics and Bioinformatics, CAMB, Gulf University for Science and Technology, Gulf, Kuwait; 4 Institute for Global Food Security, School of Biological Sciences, Queen’s University Belfast, Belfast, BT9 5DL UK; 5 Marine and Inland Waters Sciences and Technology Department, Faculty of Fisheries, Ege University, İzmir, Türkiye Turkey; 6 Department of Life and Environmental Sciences, Faculty of Science and Technology, Bournemouth University, Poole, Dorset, UK; 7 GEOMAR Helmholtz Centre for Ocean Research Kiel, Wischhofstraße 1-3, 24148 Kiel, Germany

**Keywords:** *Biological invasions*, *Economy*, *Environmental change*, *Europe*, *Society and culture*, Ecology, Ecology, Environmental sciences

## Abstract

A nexus of natural and human variables mediate the success of non-native species that threaten global biodiversity and ecological stability. However, the relative importance and interplays among relevant factors has not been holistically approached. To identify spatial differences and potential connections in relevant natural and human drivers, we analyzed the number of non-native species established in European countries using a newly collated database of established non-native species. We employ a series of broadscale national predictors classified into ‘research’, ‘economy’, ‘environment & culture’, and ‘land-use’ to predict successful establishment. Our null models, which assume the distribution of non-native species mirrors that of each predictor, accurately predicted non-native species numbers across European countries. However, a few countries were identified as outliers, having significantly over- or underrepresented non-native species numbers based on adjusted quasi-Poisson distribution quantiles. A network analysis of non-native species compositions identified these regions to be central hubs (e.g. Germany, France, and Switzerland), but also highlighted distinct spatial similarities across European countries. Combinations of the predictors ‘economy’, ‘research’, and ‘environment & culture’ explained the largest shares of differences in the number of established non-native species among European countries as well as their reporting rates over time. Individual drivers alone were insufficient to wholly explain national differences, whereas interacting driver categories ultimately accounted for the largest shares of variance. This analysis demonstrates the breadth of predictors that mediate successful establishment, and particularly highlights the relevance of overlooked historical-cultural facets affecting biological invasions.

**Supplementary Information:**

The online version contains supplementary material available at 10.1038/s41598-025-96133-0.

## Introduction

Biological invasions stemming from human activities which transport and introduce non-native species have emerged as a global burden, impacting both ecosystems and economies^1,2^. Recent assessments show that invasions are recognized as one of the major drivers of biodiversity loss, contributing to at least 60% of global extinctions alongside a myriad of more subtle ecological consequences^3,4^. The ramifications of biological invasions extend beyond environmental degradation, encapsulating substantial health risks, cultural effects, and economic repercussions^2,5^. The monetary impact of these invasions is exponentially rising and is being increasingly recognised, reflecting increasing introduction rates alongside economic development^6–8^. This trend in rising introduction rates is further exemplified by the rising number of non-native species reported annually^9,10^.

Despite these rising general trends at large scales, numerous natural and human factors influence the success of biological invasions in arriving and establishing within their new environments^11,12^. Each of these factors can mediate invasion dynamics, with the success rate of non-native species expected to fall at each stage of the invasion process^13^. Although lacking empirical support and potentially much higher in value, only a few percent of non-native species have been expected to reach the last stage of an invasion and spread, which may significantly increase their ecological or economic harm^14–16^. While the relative contributions of different factors to invasion success are debated^17^, globalization as a broad process stands out as a primary driver of species movement^18,19^, exacerbating the long-distance movement of species across biogeographic regions through enhanced trade relationships and travel^19,20^.

Central to biological invasions are therefore ‘pathways’ and ‘vectors’, referring to the routes through which non-native species are introduced into new environments (e.g. through aquaculture, shipping, or the pet trade) or, respectively, the vectors that facilitate introductions (e.g. through ballast water;^16^). Drivers are also critical, which are defined as the underlying forces or processes that facilitate, influence, or exacerbate the introduction, establishment, and spread of non-native species^21^, with the relevance of particular drivers potentially differing across invasion stages and other contexts^22^. Drivers leading to non-native species introductions are also potentially shifting over time and space, while pathways and vectors are diversifying^23,24^, for example, with e-commerce increasing the availability of exotic pet species^25^. Several socio-economic and geographic properties of recipient countries are known to mediate invasion dynamics. The investment in research, for instance, influences a country’s ability to identify non-native species and develop legislative measures to mitigate introduction rates^26,27^. Additionally, the sustainability of environmental use varies widely across countries, regions, and cultures, influenced by factors, such as land use, economic power, and historically different value systems. Ecologically, natural ecosystems with high biotic resistance could further reduce invasion success and result in declining invasion rates^28–31^. Despite growing investigations into the numerous economical, environmental, and socio-cultural factors underlying successful invasions, most scientific studies have attributed introduction rates to single, often isolated drivers^19,32,33^ or attempted holistic approaches only for single species^34^.

The complexity of biological invasions is further intertwined with major environmental drivers like climate change and human-induced habitat changes. Environmental change alters habitats and local (including climatic) conditions, potentially making ecosystems more susceptible to invasions^35^, though, not for all non-native species equally^36,37^. These drivers of invasions, however, do not act in isolation and may interact in numerous ways, challenging predictive efforts. Warmer temperatures, for instance, can allow non-native species to thrive in new areas and disrupt the balance of native ecosystems and trophic networks. Similarly, anthropogenic habitat alterations, such as urbanization, deforestation, and landscape modifications create new niches for non-native species, often leading to fragmented habitats, weakening the resilience of native species and increasing vulnerability to invasions^38–40^. As such, global hotspots of non-native species richness are linked to human activities, with trade and economic activities influencing their dispersal and establishment^17,41^ and global commerce and dispersal modulating e.g. non-native bird and plant species richness^42,43^. Understanding the dynamics of biological invasions, therefore, requires a holistic approach that considers these interdependencies. This involves integrating knowledge from economics, politics, ecology, climatology, and other disciplines, for the development of effective management strategies and biodiversity conservation in the face of changing global environmental conditions.

It is essential to effectively quantify and understand the effects of various relevant drivers using specific metrics derived from the growing availability of open data. In terms of research, indicators include biodiversity monitoring efforts, the number of researchers, and research expenditure. Economically, data points like Gross Domestic Product (GDP) or Gross National Income (GNI) per capita, the numbers of tourists and connecting airports, but also port traffic and imports are relevant as proxies of connectivity and accessibility, potentially correlating with the risk of non-native species introductions^44,45^. For environmental use, land use measures, human population density, sustainable development measures, or the ecological footprint, are pertinent, while possibly supplemented by metrics describing cultural differences, such as Trompenaars descriptors of cultural dimensions^46^. However, cultural metrics are often overlooked, but could reflect how humans perceive, interact with, and manage their environment, potentially shaping attitudes toward biodiversity, biosecurity policies, and the response to non-native species.

Given the multifaceted nature of biological invasions and the numerous of drivers involved, there is an urgent need for comprehensive studies that delve into the more complex and interrelated aspects of political dimensions (i.e. governance, *sensu* Ostrum’s social-ecological systems framework when managing non-native species^47^), but also cultural, economic, or environmental factors affecting invasions, and harness the growing availability of large-scale datasets. To this end, we extracted information on the number of established non-native species (i.e. those non-native species that have established self-sustaining populations in the wild) in European countries from a recently collated databases^45,48^ and supplemented this with national information describing differences in terms of research, the economy, environmental use and land use, which could effectively explain potential differences. Using this dataset, we aim to investigate *i*) the patterns and commonalities in established non-native species compositions and relevant predictors among European countries, and *ii*) how different predictors and their interactions influence the number of non-native species recognized as established across these countries. We hypothesize that (1) different predictors will have distinct effects on countries in terms of invasion success, which (2) will show high regional similarities. Moreover, we hypothesize that (3) neither predictor or predictor category alone can explain national differences, but that (4) their intertwined functioning – albeit differing in their importance across countries – determines the number of non-native species being reported as established.

## Results

### National composition of established non-native species

**Fig. 1:**
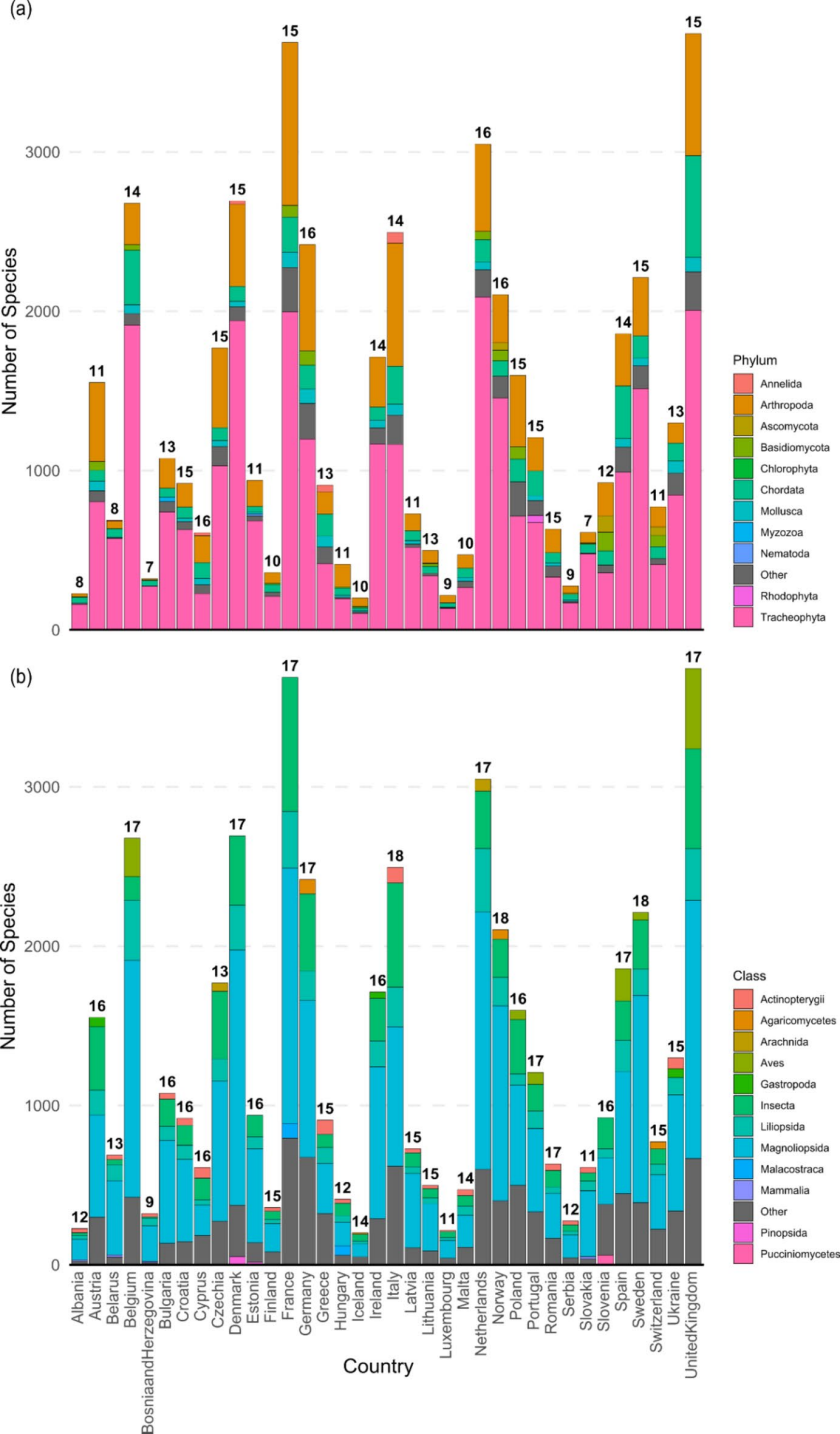
Relative composition of top-5 established non-native species phyla (**a**) and classes (**b**) among European countries. Please see Supplement 2 for a detailed overview.

Among all European countries, Tracheophyta (*n* = 8448 species; 53.41%) and Arthropoda (*n* = 3689; 23.33%) were the most species rich non-native phyla reported, followed by Chordata (*n* = 1379; 8.66%), Mollusca (*n* = 425; 2.69%), Basidomycota (*n* = 384; 2.43%), Annelida (*n* = 230; 1.45%), and Ascomycota (*n* = 228; 1.44%). All other phyla constituted < 1% of reported established non-native species. Considering classes, the most reported established non-native species belonged to the class Magnoliopsida (*n* = 6795 species; 42.97%), followed by Insecta (*n* = 2759; 17.44%) and Liliopsida (*n* = 1433; 9.06%). Aves (*n* = 681; 4.31%), Actinopterygii (*n* = 361; 2.28%), Malacostraca (*n* = 319; 2.02%), Gastropoda (*n* = 308; 1.95%), Arachnida (*n* = 298; 1.88%), and Agaricomycetes (*n* = 272; 1.72%) were the only other classes constituting > 1% of all established non-native species in our dataset (Fig. 1).

## Country-level deviations in non-native species

**Fig. 2:**
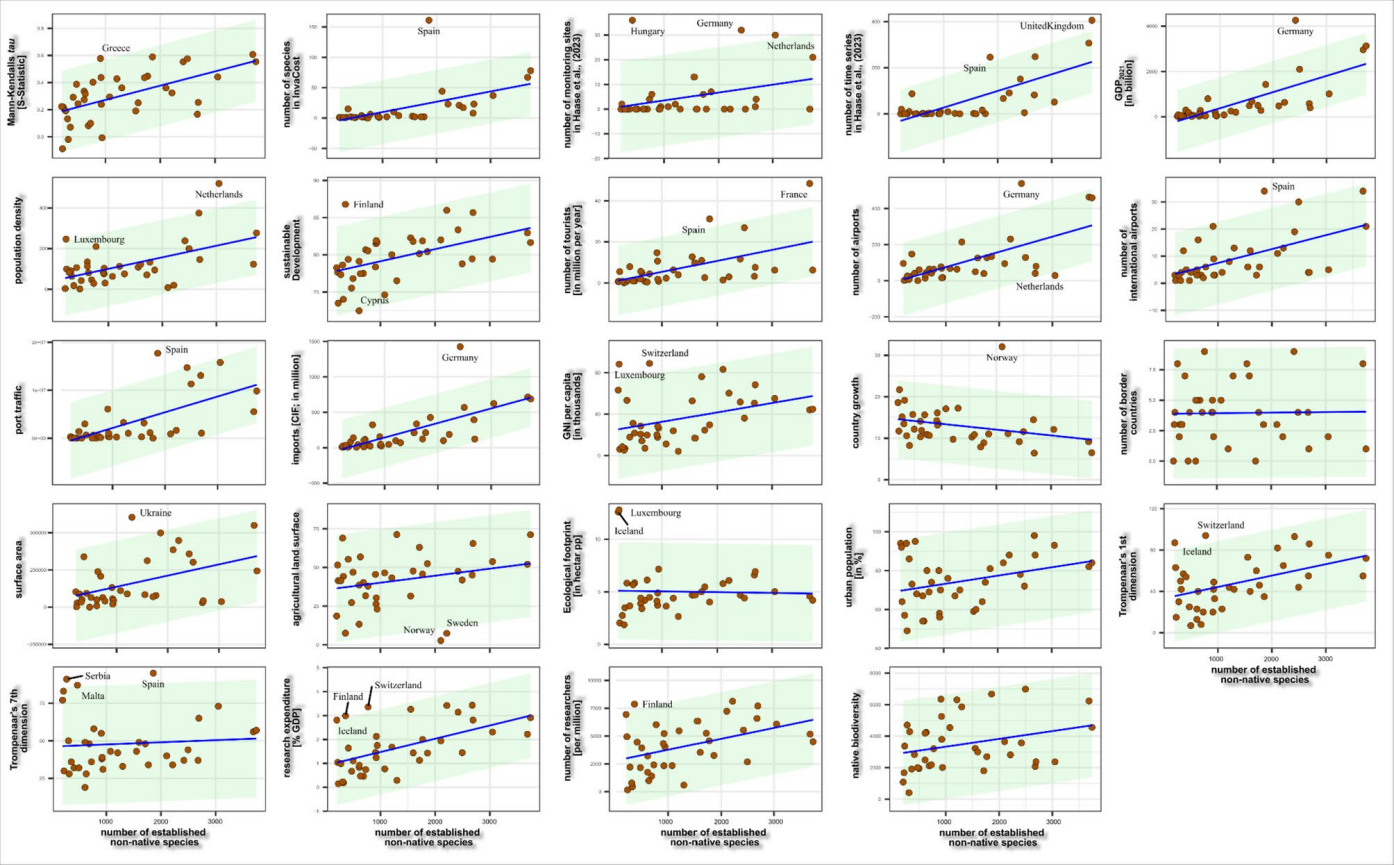
Country-level drivers of non-native species richness. Each point represents values contributed by a single country. The shaded area on each subplot reflects the expected value if the numbers of non-native species per country correspond to the respective distribution of each predictor, with the solid line a null model. Countries are named where they fall outside of this shaded interval.

Our analysis revealed distinct patterns in the relationship between established non-native species and various country-level predictors. While the majority of countries adhered to the null models produced, we identified that certain countries deviated significantly from expected values (Fig. 2), indicating over- or under-representation of non-native species given their environmental, cultural or economic conditions. Certain countries appeared frequently as outliers, including Spain, France, and Germany, however most of these outliers were underrepresented in terms of non-native species numbers. Norway and Sweden were conversely overrepresented compared to agricultural land surface, Cyprus in terms of sustainability indicators, and the Netherlands in terms of airport numbers (Fig. 2).

## Network analysis

**Fig. 3:**
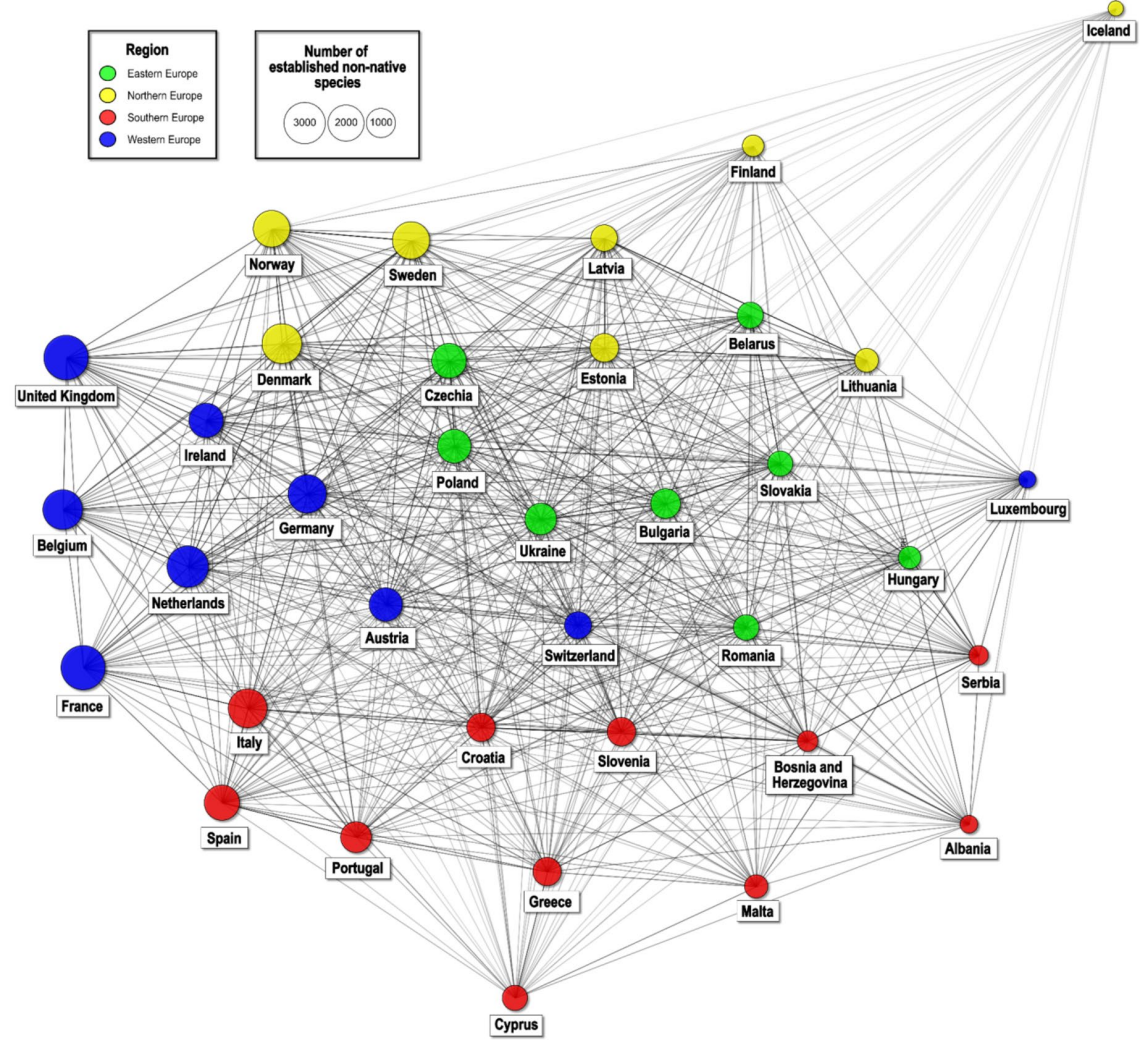
Network analysis based on the similarity in established non-native species at the country level. Nodes are scaled according to the number of established non-native species and coloured by geographic region.

The network analysis revealed clusters with denser connections indicating high similarity across Eastern, Northern, Southern, and Western European countries. Central European nations like Germany or Switzerland appeared to function as central hubs with extensive links to other countries, suggesting shared species compositions with a broad array of countries (i.e. considerable similarities), while peripheral countries such as Iceland, Malta, and Cyprus exhibit a lower degree of similarity to other countries, pointing to more distinct sets of non-native species as well as lower invasion rates (Fig. 3). Despite variations, the absence of isolated nodes indicates that all countries have some degree of similarity with at least one other country in their non-native species composition. When examining the percentage composition of classes of non-native species among countries, no discernible pattern emerged, suggesting a more complex and heterogeneous distribution of these species across different regions (Supplement 3).

## Linear modelling

**Fig. 4:**
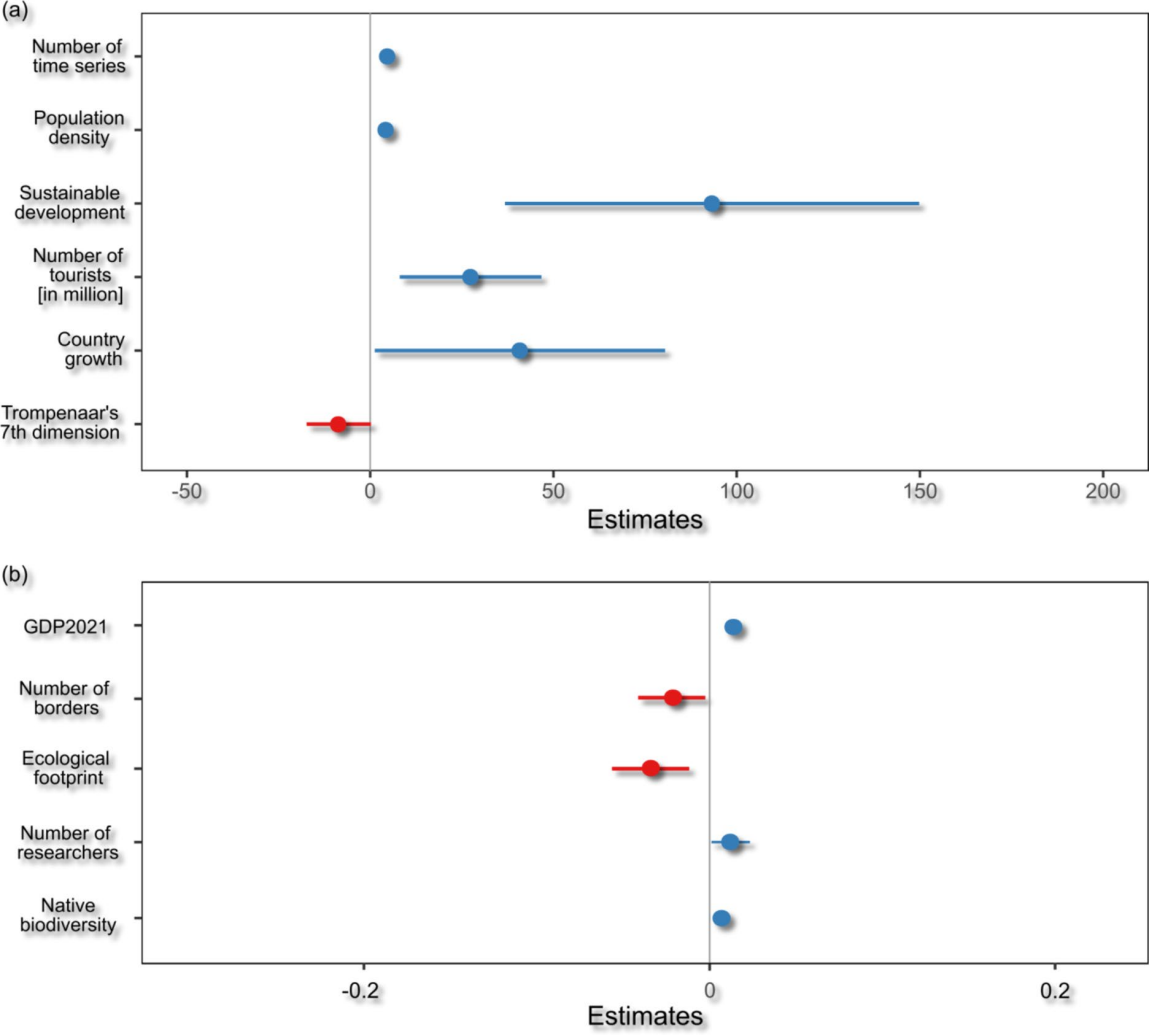
Generalized linear model results considering (**a**) numbers of established non-native species per European country and (**b**) trends in the reporting of established non-native species per country over time. Estimates on the X-axis represent the model coefficients (effect sizes) for each predictor variable, indicating the direction and magnitude of the variable’s influence on the dependent variable. Positive values suggest a positive relationship, while negative values suggest an inverse relationship between the predictor and the response variable. Detailed trajectories for (**a**) can be found in Supplement 6 and for (**b**) in Supplement 7.

Among the selected predictors, the number of long term time series, population density, sustainable development index, and the number of tourists were found to be highly significant (*p* < 0.05), whereas country growth and Trompenaar’s 7th dimension were borderline significant (*p* = 0.05–0.06; Supplement 4). A high number of long term time series, population density, sustainable development index, the number of tourists, and country growth predicted an increase in the number of known established non-native species. Trompenaar’s 7th dimension, however, indicated that a higher value (i.e. cultures that tend to strive to take control) resulted in lower numbers of established non-native species (Fig. 4a).

From the five predictors identified as relevant for the trend in the reporting of established non-native species, all were found to be significant (*p* < 0.05) except for the number of researchers (*p* = 0.075; Supplement 5). The strength of the incline in established non-native species being reported over time was positively predicted by GDP, the numbers of researchers, and native biodiversity. Both the number of border countries and the ecological footprint predicted a slower increase in non-native species reporting (Fig. 4b).

## Variance partitioning

**Fig. 5:**
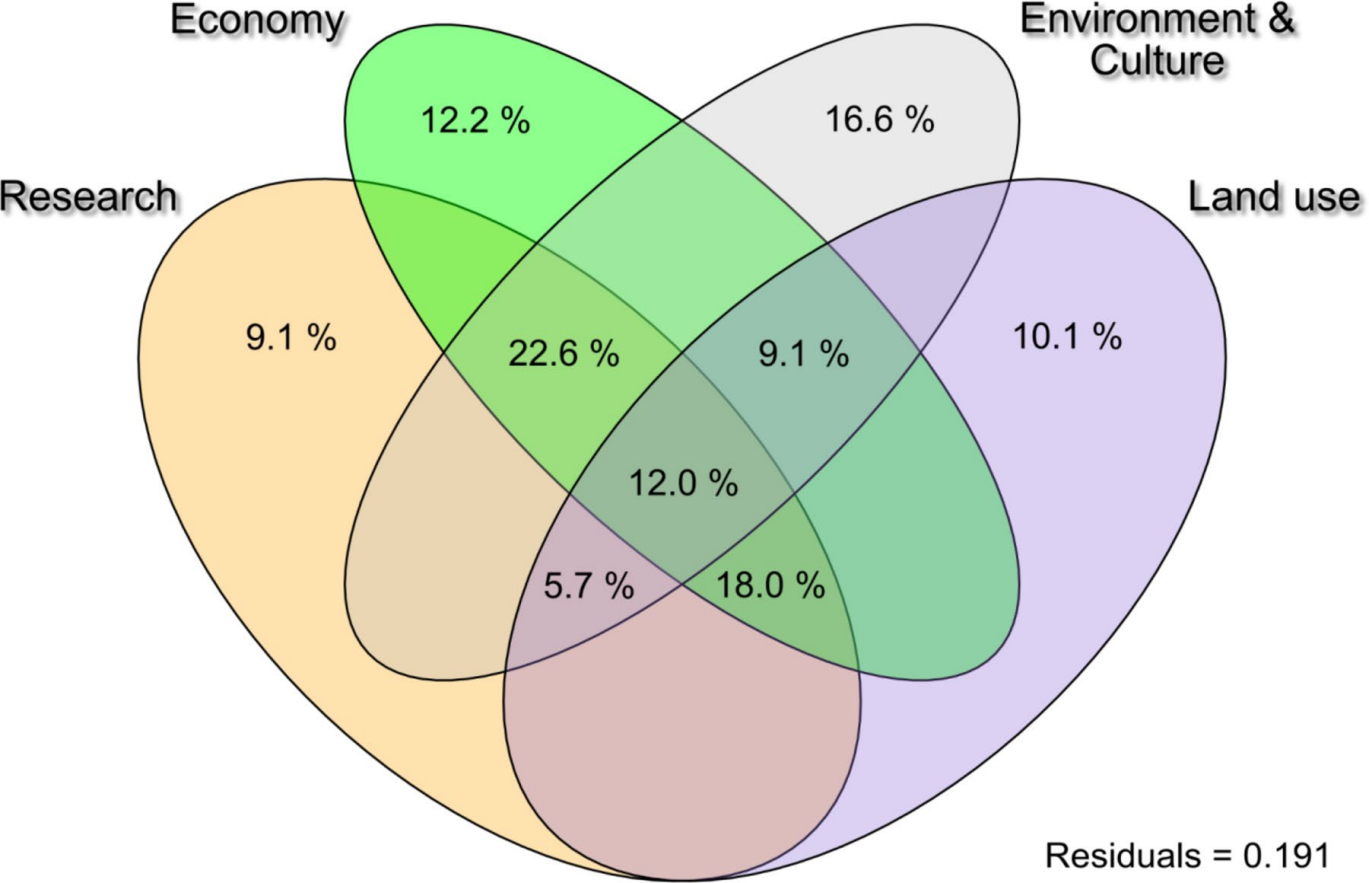
Results of the variance partitioning (relative contribution of predictor classes; in %) for predictors classified under the umbrellas “research”, economy”, “environment & culture”, and “land use”. Only predictor classes and combinations > 2% are displayed.

Economic predictors alone explained 12.2% (*p* < 0.05), while research effort (i.e. the number of macroinvertebrate long term time series) explained 9.1% (*p* < 0.05). Predictors describing environmental or cultural norms explained 16.6% (*p* < 0.05), and land use explained 10.1% (*p* < 0.05). The highest explanatory power had a combination of economic, research, and environmental/cultural norms with 22.6%, followed by 18.0% for a combination of economic, research and land use (Fig. 5).

## Discussion

Biological invasions culminate as a result of interacting human and natural phenomena that are difficult to disentangle^49,50^. While the establishment of non-native species is driven by finer-scale mechanisms rather than national-level predictors, the total number of established non-native species in a country is likely influenced by co-occurring drivers, rather than single predictors. Hence, rather than disentangling predictors influencing the establishment process itself, we show that several socio-economic, cultural, and environmental predictors are predictive of the reporting of established non-native species at the country-scale in Europe. We further identify predictors which are primary drivers of invasion rates and trends, with relatively balanced contributions across economic, environmental, land use, and research elements. However, economic, research, and environmental/cultural terms when combined explained the largest share of variance, indicating the breadth of factors which affect invasions. We further highlight spatial patterns of country clustering, showing that those which are geographically close have a higher similarity in non-native species composition, potentially signaling the importance of proximity and bridgehead effects (where an initially established non-native population acts as a secondary source for further invasions, facilitating spread into new regions) for future spread.

## Outliers and networks among countries

Most countries tended to fall within the expected numbers of non-native species. This trend could reflect our focus on European countries, where several variables like ecological footprint and population density can show relatively little variation among states. The more frequent appearance of countries such as Spain, France, and Germany as underrepresented outliers in terms of the numbers of reported established non-native species may be attributed to a variety of complex, interrelated links and factors that extend beyond the considered predictors. For instance, Germany’s status as an outlier in terms of GDP is directly linked to its outlier status for Cost, Insurance, and Freight (CIF) imports, highlighting a strong correlation between economic output and the volume of imported goods^51^. Similarly, the high levels of tourism in France and Spain, along with Spain’s extensive number of airports and ports, underscore the significant role of tourism and transport infrastructure in influencing these countries’ economic and environmental profiles. Countries are not homogenous in nature^52^, with considered predictors arguably spatially differing within the countries with larger surface area, which we did not directly account for. In contrast, countries such as Norway and Sweden were overrepresented for some predictors, particularly agricultural land surface, indicating that colder regions with lower agricultural dominance are still highly vulnerable to invasions. This geographic but also the climatic, economic, and environmental diversity within these countries could contribute to a varying capacity for detection and reporting of non-native species, resulting in countries being identified as outliers and invasions clustering at the subnational scale. These patterns are likely facilitated by differing scientific communities, policy and regulatory frameworks^53,54^, as well as surveillance systems for detecting non-native species^34^.

The observed patterns in the outlier and network analysis, particularly the clustering of countries based on the similarity in reported established non-native species, could be attributed to similar environmental conditions, but also several ecological and anthropogenic factors. Indeed, due to the matching ecological requirements that favor survival and reproduction^55^, similar environmental conditions and habitats can result in ecologically similar non-native species successfully establishing. These factors were not accounted for in the analyses because climatic conditions, such as Köppen-Geiger climate zones^56^, extend beyond national boundaries and can differ significantly even within countries^57^. While climatic or environmental conditions determine which non-native species could potentially establish^58^, other potential predictors like propagule pressure are considered more relevant in determining actual establishment success^59^.

Communities of successfully established non-native species are the outcome of a variable interplay of various other factors, ultimately shaping patterns and similarities. The centrality of some European countries in the network likely reflects major transport and trade routes^60,61^, as countries have different levels of international trade and tourism, i.e. connections with each other, which are known pathways for the introduction and spread of non-native species^44^ The volume and diversity of goods and people moving across borders also increase the chances of species being accidentally introduced and becoming established. For example, the United Kingdom has previously been identified as a central hub for biological invasions^62^, whereas central European countries like Germany may act as ‘hub’ locations due to their dense road, rail, and river networks facilitating the movement of goods and people^63^, along with the associated species^45^. This aligns with the concept of ‘bridgehead effects’, as well as shared risks that emerge along international borders, such as between Norway and Sweden^64^. Therefore, central or well-connected regions in Europe could become hubs for biological invasions which rapidly radiate across neighboring countries and lead to the observed clustering.

The peripheral positioning and disconnection of countries like Iceland, Finland, and Cyprus can be conversely explained by their insularity and lesser degree of land-based connectivity with the rest of Europe. This isolation might limit the variety of non-native species that can be established, as these countries are less exposed to the continual influx of species from the mainland. In contrast, it is possible that their isolation increases invasion success and impacts, particularly in the context of a changing climate^65^. Luxembourg is a particular case that, despite being one of the smallest countries in Europe, is a major centre for banking and finance. Combined with its cross-border dynamics and epicentre of European policies, Luxembourg is a unique country with Europe whose relationship seems more aligned with Hungary, Lithuania, and Serbia, rather than its geographical neighbours. This could be driven by shared trade routes, similar levels of urbanization, or common agricultural practices, but could also include similarities in habitat types, climate conditions, or shared pathways of introduction (e.g. through specific trade goods or landscaping plants) that are not as prevalent in its immediate geographical vicinity. Additionally, the presence of specific policies or environmental conservation practices in Luxembourg and these countries could play a role in determining the types of non-native species that successfully establish – possibly linking to differences in the stringency at which legislation is implemented^66^.

## Predictors of invasion rates

Our results corroborate that biological invasions are modulated by a complex interplay between environmental, economic, and socio-cultural factors rather than isolated predictors^11,12^. We have included several predictors and combinations that had been previously overlooked with existing data resources. The significant predictors modulating the number of reporting established non-native species and their reporting rates over time across European countries underline the importance associated with long-term biomonitoring efforts (as proxied by the number of time series) and human population density with an increased number of known established non-native species. However, although these findings could also suggest that the reported number of established non-native species in most countries is poorly inventoried, these findings highlight the critical role of human observation (i.e. applied scientific efforts and citizen reporting) in detecting establishment non-native species. Sustainable development and the influx of tourists reflect the extent of human activity and mobility that can inadvertently introduce non-native species into new environments. Similarly, the economic growth of a country may increase its connectivity and trade, further elevating the risk of species introduction. Moreover, Trompenaar’s 7th dimension (which contrasts cultures that tend to dominate their environment against those that prefer to coexist with it), was found to be relevant in predicting biological invasions. This metric introduces a particularly new and nuanced understanding of how societal attitudes towards nature can influence biodiversity outcomes^67,68^. A higher value on this dimension, indicating a ‘control-oriented’ approach, may lead to more stringent environmental regulations and biosecurity measures, effectively reducing the number of established non-native species or increasing their detection rate, suggesting that cultural predispositions towards controlling or adapting to the environment can significantly impact the likelihood of non-native species establishment and require further investigation.

Predictors for the rate at which non-native species are reported over time, including the national GDP, the number of researchers, and native biodiversity, differed and underscore the importance of economic resources and scientific capacity in detecting and documenting biodiversity changes. A higher GDP and more researchers indicate greater financial and intellectual resources dedicated to environmental monitoring and research, which can enhance the long-term detection and reporting of non-native species^69,70^. The positive effect of biodiversity is contrary to the biotic resistance hypothesis^71^, but could be a marker for greater environmental awareness to report invasive species, positive effects of pre-adaptation and ecological similarity, or a greater amount of available niches, since larger countries with more native species likely support more non-natives. The negative correlation between the number of border countries and ecological footprint with the rate of non-native species reporting, contrary to expectations, suggests that higher levels of environmental disturbance do not always correspond with increased invasion rates. Borders have been identified as high-risk areas for biological invasions by vertebrates^64^, but most countries here had high numbers of established non-natives irrespective of border prevalence in mainland Europe. This could imply that countries with a higher ecological footprint, despite their potentially greater impact on the environment, may also be more aware and proactive in their monitoring and reporting efforts, or could have more financial resources to support management. This awareness and action could stem from a recognition of the risks associated with biodiversity loss and the need to manage non-native species more effectively, reflecting a complex relationship between environmental degradation and conservation efforts.

There is, however, a temporal mismatch between the timing of non-native species invasions that mostly occurred in the past and the explanatory variables used in our analysis reflecting more recent conditions. This is mitigated to some extent because many of the national predictors, such as GDP, population density, and cultural dimensions, have demonstrated long-term consistency in their rates of change. These predictors, when compared among European countries, provide a reasonable approximation of past conditions, whereas retrospective analysis using current data can still offer valuable insights into historical patterns and drivers of non-native species establishment (respectively, their reporting). While acknowledging the temporal mismatch between invasions and explanatory variables, our approach remains valid for understanding the broader patterns and drivers of non-native species establishment.

### Driver combinations

Our multifactorial analysis found that most single and combined groupings contributed to invasion rates. The distinct contributions of economic predictors, research efforts, environmental or cultural norms, and land use patterns to the observed variance highlight that no single category of drivers can fully explain the complex dynamics of non-native species establishment and proliferation. Notably, the combination of economic, research, and environmental/cultural norms, demonstrating the highest explanatory power (22.6%), suggests that the interactions between these predictors are critical in shaping biodiversity outcomes^72^. This implies that the economic context of a country, such as its level of wealth and investment in trade and development, intersects with its commitment to research and monitoring efforts, which in turn is influenced by cultural attitudes towards environmental conservation and management^73,74^.

Moreover, our results highlight the relevance of often neglected cultural differences. The significant explanatory power of environmental or cultural norms alone (16.6%) points to the deep-rooted influence of societal values and practices on ecological processes. These norms can dictate the level of priority given to environmental issues, including the management of non-native species, reflecting political willingness to allocate resources towards conservation efforts^75,76^. Additionally, the role of land use changes, which include agricultural expansion, urbanization, and habitat modification, underscore the impact of human alterations to the landscape on biodiversity patterns, particularly in facilitating or hindering the spread of non-native species.

### What drives biological invasions and introduction rates?

Understanding the drivers behind introduction rates and ultimately successful biological invasions entails considering complex historical legacies, contemporary trade dynamics, and spatially differing yet evolving economic, social, and cultural human activities^77^. Historically, trade routes and patterns have had particularly significant influence over introduction rates, shaping present-day dynamics^10,78^. Rapidly growing sectors such as horticulture, aquaculture, fisheries, and the trade in aquarium and pet species are increasingly contributing to species introductions^20,25,79^. Compounding this complexity is the spatial and temporal diversity exhibited by countries, which undergo cultural, economic, and environmental transitions over time. It is therefore crucial to recognize the fluctuating nature of relevant drivers temporally and the difficulty in weighing their relative importance at the national level, though attempting to precisely explain past differences may prove challenging.

These aspects, together with the often questionably assumed correlation between introduction rates, the successful establishment of non-native species, and invasiveness^80^, makes predictive efforts and national deny- or “black”-lists challenging to implement^81,82^. Nonetheless, national assessments and lists aiming to minimize the threat of invasive species hold critical importance for stakeholders and policymakers, given that countries represent the smallest relevant political *units of action*^83,84^. Such lists will, however, require further improvement and predictive strength and thus, efforts should be redirected towards preventing future invasions. By understanding the multifaceted nature of invasion dynamics and acknowledging the temporally fluctuating nature of relevant drivers, stakeholders and policymakers can adopt more effective strategies for managing and conserving ecosystems and promoting sustainable socio-economies.

### Methods

#### Data

To investigate drivers determining the number of reported established non-native species, we used data recently published by Henry et al.^45^ which integrated data from the the SInAS workflow of non-native species occurrences^85^ with data from the ‘Theory and Workflows for Alien and Invasive Species Tracking’ (sTWIST;^7^) and^86^. We excluded species without assigned invaded country/region or those not reported in the European Union. Additionally, species listed only as CASUAL and ABSENT in the ‘degreeOfEstablishment’ and ‘occurrenceStatus’ columns were removed due to their unclear establishment status^87^. Species identities and scientific names were verified against the Global Biodiversity Information Facility^88^. If a species was not found in this database, we conducted opportunistic internet searches in December 2023 using www.google.de and www.google.scholar.com to confirm its authenticity and, finally, corrected misspelled names verified against GBIF and removed any duplicate entries from the dataset. Distinguishing between introduced and established non-native species presents a challenge, as ‘introduced’ pertains to the species level, whereas ‘established’ reflects population-level dynamics. Since multiple populations of the same species can exist at different stages of the invasion process and shift over time^80^, classification discrepancies may arise. Consequently, we recognize the potential for outdated or erroneous entries—such as misclassifications of species status—to have been carried over from the original sources^7,85,86^ to^89^, despite efforts to identify such inconsistencies during manual validation.

### Analysis

To infer variability in the rate at which established non-native species reported across the different European countries over time of, we used the temporal information on the national reporting of established non-native species in sTWIST by year. With this data, we computed for every country the Mann-Kendall’s tau ($\tau$) and the respective variance in the annual reporting of established non-native species^90^ using the *mmkh* function from the modiedmk R package^91^. In this analysis of raw annual reportings, $\tau$ presents a monotonic trends’ S-statistics (i.e. slope;^92^) for the reported non-native species over time-based^10^.

To identify national differences that could contribute both to the total number of reported established non-native species as well as rates at which established non-native species were reported over time, we selected a series of broadscale national predictors (see Table 1 for an explanation and reasoning of each predictor and Supplement 1). Some variables, like the number of researchers and research expenditure, serve as proxies for historical research capacity and awareness, influencing both past and present monitoring and reporting of non-native species, whereas others, like surface area or the number of border countries, were included to account for potential exposure and accessibility to non-native species. Each economic predictor—GDP representing overall economic output, GNI per capita illustrating average citizen income, and country growth reflecting changes in economic performance over time— as well as predictors describing economic trade volume and capacity, were incorporated to capture various facets of a nation’s economic landscape. By examining these distinct elements, we aimed to provide a holistic view of how economic predictors affect long-term biomonitoring efforts. Additionally, these predictors were selected for their ability to reflect both the financial capacity and cultural context essential for sustaining long-term ecological monitoring. This approach enables us to evaluate not only the availability of infrastructure but also the nation’s underlying commitment to biomonitoring and ecological research. Drawing from extensive cross-cultural research conducted in the early 1990s, Trompenaars’ first and seventh cultural dimensions—universalism versus particularism and sequential versus synchronic time^67,93^—were identified as possibly relevant cultural predictors to assess their impact on the development of sites belonging to the European Long-Term Ecological Research Network (eLTER;^94^). These dimensions were chosen due to their importance in interpreting how diverse cultural perspectives on rules and time management can affect collaborative scientific projects across various regions.

These were grouped into four categories, namely ‘research’, ‘economy’, environment & culture’, and ‘land use’ predictors (Table 1). While land use is an environmental measure, it was treated as a separate predictor category to emphasize its direct human-driven modifications to landscapes, such as agricultural expansion and urbanization, which influence invasion dynamics differently from broader environmental and cultural predictors. This distinction allows for a clearer assessment of its independent contribution to non-native species establishment, owing to the importance of ecological disturbance for invasion success^95^. There is however a potential temporal mismatch between the timing of invasions and the national predictors reflecting recent conditions, but arguably their long-term consistency and gradual changes make them reasonable approximations of historical conditions.

**Table 1:** List of considered predictors, their units, explanation, and source, categorized (Cat) into ‘research’ (A), ‘economy’ (B), ‘environment & culture’ (C), and ‘land use’ predictors (D).

Predictor	unit	Cat	Explanation	origin/reference
the number of macroinvertebrate long term time series	individuals	A	a proxy for biomonitoring efforts	^96^
research expenditure as percentage of annual GDP	%	A	the percentage of GDP spent on research and development	*data.worldbank.org*
the number of researchers		A	the count of professionals conducting research	*data.worldbank.org*
the gross domestic product of 2021 (GDP_2021_)	in millions	B	the total value of goods and services produced in a country in 2021	*statista.com*
the number tourists (2021)	million	B	total international visitor arrivals in the year 2021	*worlddata.info*
the number of airports		B	total count of facilities for aircraft takeoff and landing	*cia.gov/the-world-factbook/field/airports/country-comparison/*
the number of international airports		B	count of airports with customs and immigration for international travel	*en.wikipedia.org/wiki/List_of_international_airports_by_country;* http://www.transtats.bts.gov/Data_Elements.aspx
port traffic	in twenty-foot equivalent unit (TEU)	B	the number of ships cargo arriving and departing from a country’s ports	https://data.worldbank.org/indicator/IS.SHP.GOOD.TU?most_recent_value_desc=true (TEU)
import volume	cost, insurance, and freight (CIF); in millions	B	the total value or volume of foreign goods and services purchased	*wits.worldbank.org*
gross national income (GNI) per capita	US$ millions; Atlas method (current US$)	B	total national income divided by the human population	*wits.worldbank.org*
country growth	in US$ million	B	annual percentage growth rate of the country’s trade value (export or import), by sector, at market prices in current U.S. dollars	*wits.worldbank.org*
the number of border countries/shared borders		B	the count of countries adjacent to a nation’s borders	*cia.gov*
population density	people per km²	C	the number of people per unit area in a region	*macrotrends.net*
the sustainable development index		C	a composite measure of a country’s economic, social, and environmental progress	^97^
ecological footprint	in hectares per capita	C	human demand on natural resources relative to ecosystem capacity	*worldpopulationreview.com/country-rankings/ecological-footprint-by-country*
Trompenaar’s 1st cultural dimension	universalism vs. particularism	C	describes the ethical dilemma between applying general rules universally or adapting decisions based on specific circumstances and relationships	www.thtconsulting.com/culture-factory/culture-explore/compare-countries/
Trompenaar’s 7th cultural dimension	internal direction vs. outer direction	C	contrasts the belief that individuals can control their environment and destiny with the belief that external forces or fate control the environment and destiny	www.thtconsulting.com/culture-factory/culture-explore/compare-countries/
known native biodiversity		C	the diversity of native species in an ecosystem	*worldrainforests.com*
country surface area	km²	D	the total land and water area of a country	*ec.europa.eu/eurostat/statistics-explained/index.php? title = Land_cover_statistics#Land_cover_in_the_EU_Member_States*
percent of surface being agricultural land	%	D	proportion of land used for farming	*data.worldbank.org/indicator/AG.LND.AGRI.ZS? locations = BE*
percentage of population living in urban areas	%	D	proportion of people living in cities	*data.worldbank.org/indicator/SP.URB.TOTL.IN.ZS? locations = EE-FI-FR*

### Identifying National outliers

To statistically identify outliers (i.e. countries that are over- or under-represented) among predictors related to the number of established non-native species per country used in subsequently conducted analyses, we built a series of null models for each predictor^89,98^. These linear models were based on the premise that the distribution of established non-native species across countries mirrors the distribution of each predictor. Our approach entailed generating a defined region around the null model to represent the probability of a predictor being significantly over- or under-represented, as evidenced by a country’s placement inside or outside of this region. A predictor is over- or under-represented if the observed number of non-native species in a country is significantly higher or lower than expected based on that predictor’s value. This indicates a deviation from the expected relationship suggesting that the predictor does not fully explain the variation in non-native species numbers, potentially due to additional factors or limitations in its explanatory power as neither predictor alone can explain national differences. The region’s boundaries were determined using the upper and lower quantiles of a quasi-Poisson distribution, adjusted for multiple comparisons via a Bonferroni correction. Specifically, we aimed for (1-α/m) × 100% of the distribution to fall within these boundaries, where α represents the significance level set at 0.05, and m is the number of established non-native species in a given country. This adjustment ensures that the statistical threshold for identifying outliers becomes more stringent as the number of species increases, thus controlling for the false discovery rate. Our analysis was restricted to European countries with at least one reported established non-native species.

### Network analysis

To identify similarities in the composition of established non-native species among European countries and subsequently to identify central nodes (i.e. countries) in this network, we used a network analysis to visualize the interconnections among European countries based on the number and identity of shared established non-native species at the European scale. For this, we utilized the vegan R package^99^ to generate a Jaccard-based similarity matrix and constructed a bipartite network using the igraph R package^100^ where nodes are represented by countries while the link between countries is represented by the similarity (based on the number of shared established non-native species). Consequently this means that the closer countries group together, the more similar they are in terms of established non-native species assemblages.

### Multivariate model selection and analyses

To assess correlations among predictors and to test for multicollinearity^101^, we employed the variance inflation factor (VIF) analysis using the vif function from the R package car^102^. This analysis led to the exclusion of four predictors: number of airports, number of international airports, import volume, and research expenditure as a percentage of annual GDP, due to their VIF values surpassing the threshold of 10, indicating high multicollinearity. Subsequently, we applied a stepwise (forward) model selection for two distinct models using the glmulti package in R^103^. The first model selection (m1) aimed to elucidate the predictors influencing the reported number of established non-native species per country. Through the model selection process, relevant predictors were identified as: [m1.1] the number of time series, [m1.2] population density, [m1.3] the sustainability index, [m1.4] the number of tourists, [m1.5] country growth, and [m1.6] Trompenaar’s 7th dimension. The second model selection (m2) focused on understanding variability in the reporting of established non-native species over time. For this model, the model selection highlighted [m2.1] GDP, [m2.2] the number of borders, [m2.3] the ecological footprint, [m2.3] the number of researchers, and [m2.4] native biodiversity as significant predictors.

For both models, we chose generalized linear models with negative binomial distribution implemented in the glm function of the stats R package^104^. The first model consisted of the number of established non-native species as a response variable as well as the as relevant identified predictors (m1.1 – m1.6). The second model consisted of each country’s respective trend’s S-statistic in the establishment of non-native species and the as relevant identified predictors (m2.1 – m2.4). We tested for overdispersion in both models using the dispersion test to confirm the suitability of the negative binomial distribution over the standard Poisson model. To check for potential overfitting, we implemented k-fold cross-validation, ensuring that the models maintained predictive accuracy and generalizability. Finally, model diagnostics included residual analysis and the assessment of influential data points to confirm that the models were well-calibrated and not unduly influenced by individual observations, providing robust and reliable interpretations of the data. This methodical approach ensured that both models were optimized to accurately reflect the underlying patterns and influences in the data.

### Variance partitioning of different driver classes

To investigate the predictors that influence the reporting of established non-native species and how their reporting trends over time differ across European countries, we utilized a variance partitioning model designed to dissect the contribution of different explanatory variables or groups of variables to the observed variance in a dependent variable^105^. This analysis was conducted using the varpart function implemented in the *vegan* R package. The varpart function is adept at quantifying the unique contribution of each category of drivers (i.e. drivers classified belonging to “research”, “economy”, “environment & culture”, and “land use”; see Table 1) by reporting the adjusted R² value for each. The adjusted R² value is particularly informative as it accounts for the number of explanatory variables involved, thus providing a more accurate measure of the explanatory power of each driver category. This adjustment is critical for ensuring that the significance of the variables is not merely a function of their number, thereby allowing for a fair comparison across different sets of variables. To further validate the significance of the contributions made by each group of drivers, we applied the anova.cca function from the *vegan* package. This function performs a series of permutation tests (*n* = 10,000) to assess the statistical significance of each group of variables.

## Electronic supplementary material

Below is the link to the electronic supplementary material.

Supplementary Material 1

## Data Availability

Data analysed in this manuscript is provided as Supplementary Material.
