# Supplementary material for: Analysing factors underlying the reporting of established non-native species
Source: Sci Rep. 2025 Apr 10;15:12337. doi: 10.1038/s41598-025-96133-0 (PMC11985998; doi:10.1038/s41598-025-96133-0)

**Supplementary Material**

**Supplement 1.** Data used in this manuscript

| Country | Elzas_DB | InvaCost | Haase | n_timeseries | GDP2021_b | Pop_dens | Sust_development | tourists_m | n_airports | n_airports_intl | ports_traffic | imports_CIF_in_million | GNI_per_capita_in_thousands | Country_Growth | n_border_countries | Surface_area | Agricultural_land | Ecological_footprint_hectat_pp | urban_pop_perc | Trompenaar_1 | Trompenaar_7 | Research_expenditure_perc_GDP | Researcher_pp | Nat_Biodiv | tau | p_value | Group_region | Group_culture |
| --- | --- | --- | --- | --- | --- | --- | --- | --- | --- | --- | --- | --- | --- | --- | --- | --- | --- | --- | --- | --- | --- | --- | --- | --- | --- | --- | --- | --- |
| Albania | 228 | 1 | 0 | 0 | 18,26 | 99.29 | 73.51 | 5.52 | 3 | 2 | 145672 | 7.735 | 6.100 | 21.71 | 4 | 28748 | 41.5 | 2.05 | 64 | 15 | 30 | 0.15 | 158 | 3369 | 0,219610319 | 0,017690539 | Southern Europe | Latin Europe |
| Austria | 1553 | 3 | 13 | 2 | 489,4 | 108.59 | 82.28 | 12.73 | 50 | 6 | 285900 | 210.913 | 52.760 | 10.28 | 8 | 83878 | 31.5 | 4.7 | 59 | 73 | 43 | 3.26 | 6342 | 3225 | 0,189995319 | 0,000188828 | Western Europe | Germanic Europe |
| Belarus | 689 | 1 | 0 | 0 | 68,21 | 46.14 | 77.50 | 0.787 | 65 | 3 | 0 | 41.811 | 7.040 | 15.64 | 5 | 207600 | 40.3 | 4.20 | 80 | 8 | 48 | 0.46 | 1394 | 2115 | 0,081555583 | 0,633322954 | Eastern Europe | Slavic Europe |
| Belgium | 2678 | 8 | 1 | 82 | 584,1 | 375.0 | 79.46 | 3.24 | 41 | 4 | 13075891 | 393.655 | 50.530 | 14.45 | 4 | 30666 | 45.1 | 6.6 | 98 | 55 | 37 | 3.43 | 6582 | 2077 | 0,16601944 | 0,000371814 | Western Europe | Germanic Europe |
| BosniaandHerzegovina | 322 | 1 | 0 | 0 | 23,37 | 63.8 | 74.02 | 0.502 | 24 | 4 | 0 | 13.029 | 6.900 | 19.15 | 3 | 51209 | 44.2 | 3.49 | 49 | 42 | 28 | 0.19 | 443 | 4286 | -0,019926408 | 0,928422689 | Southern Europe | Slavic Europe |
| Bulgaria | 1077 | 2 | 1 | 9 | 84,06 | 63.32 | 74.62 | 2.3 | 68 | 4 | 245160 | 46.396 | 11.200 | 17.1 | 5 | 110.996 | 46.5 | 3.64 | 76 | 23 | 43 | 0.77 | 2339 | 4543 | 0,294106722 | 0,0000398159 | Eastern Europe | Slavic Europe |
| Croatia | 920 | 2 | 0 | 0 | 68,96 | 75.8 | 81.50 | 10.64 | 69 | 9 | 367646 | 33.735 | 17.630 | 16.12 | 5 | 56.593 | 26.4 | 3.72 | 58 | 42 | 38 | 1.24 | 2331 | 5251 | 0,437401861 | 0,0000414848 | Southern Europe | Slavic Europe |
| Cyprus | 611 | 4 | 0 | 2 | 28,41 | 134.49 | 72.49 | 1.94 | 15 | 3 | 319131 | 10.231 | 28.470 | 14.09 | 0 | 9.252 | 13.3 | 3.88 | 67 | 13 | 19 | 0.83 | 1743 | 2489 | 0,336038381 | 0,00000452995 | Southern Europe | Latin Europe |
| Czechia | 1770 | 2 | 0 | 1 | 281,8 | 133.14 | 81.87 | 2.6 | 128 | 6 | 0 | 212.481 | 24.530 | 8.89 | 4 | 78.871 | 45.7 | 5.47 | 74 | 46 | 34 | 2 | 4569 | 2696 | 0,447729379 | 0 | Eastern Europe | Slavic Europe |
| Denmark | 2693 | 23 | 4 | 248 | 398,3 | 146.45 | 85.68 | 7.59 | 80 | 4 | 1055262 | 121.784 | 68.300 | 6.41 | 1 | 42.925 | 65.5 | 6.93 | 88 | 86 | 65 | 2.81 | 7708 | 2373 | 0,253672093 | 2,98023E-06 | Northern Europe | Germanic Europe |
| Estonia | 939 | 1 | 1 | 10 | 37,19 | 30.61 | 81.68 | 0.808 | 18 | 3 | 277000 | 24.203 | 26.460 | 13.51 | 2 | 45.336 | 23.1 | 7.16 | 70 | 33 | 31 | 1.75 | 4038 | 2008 | -0,007091971 | 0,907827258 | Northern Europe | Baltic States |
| Finland | 360 | 15 | 0 | 10 | 297,3 | 18.25 | 86.78 | 0.807 | 148 | 12 | 1409037 | 82.264 | 53.280 | 10.61 | 3 | 338.411 | 7.5 | 5.83 | 86 | 57 | 36 | 2.99 | 7871 | 1912 | 0,07062538 | 0,380552173 | Northern Europe | Nordic Countries |
| France | 3688 | 67 | 0 | 307 | 2958 | 123.27 | 82.95 | 48.4 | 464 | 34 | 5522541 | 714.842 | 44.160 | 9.17 | 8 | 549.060 | 52.1 | 4.60 | 82 | 55 | 56 | 2.22 | 5175 | 6236 | 0,607649922 | 0 | Western Europe | Latin Europe |
| Germany | 2419 | 21 | 32 | 151 | 4260 | 238.55 | 83.36 | 11.69 | 539 | 19 | 14716437 | 1424.675 | 51.660 | 9.15 | 9 | 357.569 | 47.5 | 4.7 | 78 | 93 | 37 | 3.14 | 5536 | 3571 | 0,553774774 | 0 | Western Europe | Germanic Europe |
| Greece | 909 | 4 | 0 | 0 | 214,9 | 82.55 | 78.37 | 14.71 | 77 | 21 | 6069480 | 154.570 | 20.000 | 14.24 | 4 | 131.694 | 45.5 | 4.12 | 80 | 20 | 55 | 1.46 | 4326 | 6348 | 0,577918947 | 0 | Southern Europe | Latin Europe |
| Hungary | 412 | 2 | 36 | 87 | 181,8 | 106.64 | 79.39 | 7.93 | 41 | 5 | 0 | 139.132 | 17.780 | 8.25 | 7 | 93.012 | 55.3 | 3.67 | 73 | 54 | 32 | 1.64 | 4452 | 2830 | 0,291558176 | 0,000492692 | Eastern Europe | Central Europe |
| Iceland | 201 | 1 | 0 | 0 | 25,6 | 3.6 | 78.27 | 0.698 | 96 | 3 | 297263 | 7.838 | 63.140 | 18.6 | 0 | 103000 | 18.6 | 12.6 | 94 | 87 | 77 | 2.81 | 6940 | 1088 | 0,222831994 | 0,000633717 | Northern Europe | Nordic Countries |
| Ireland | 1713 | 2 | 6 | 16 | 504,2 | 70.95 | 80.15 | 10.95 | 40 | 3 | 1143007 | 122.755 | 76.110 | 7.89 | 0 | 69.947 | 63 | 5.01 | 64 | 60 | 48 | 1.13 | 5251 | 1802 | 0,436086446 | 0 | Western Europe | Germanic Europe |
| Italy | 2495 | 17 | 0 | 5 | 2100 | 199.89 | 78.79 | 26.89 | 129 | 30 | 11303247 | 568.202 | 36.130 | 11.59 | 4 | 302.072 | 41.9 | 4.41 | 72 | 44 | 44 | 1.45 | 2678 | 6984 | 0,575848341 | 0 | Southern Europe | Latin Europe |
| Latvia | 729 | 0 | 4 | 3 | 39,85 | 29.01 | 80.68 | 0.478 | 42 | 2 | 434388 | 23.086 | 19.830 | 10.91 | 4 | 64.585 | 31.7 | 6.13 | 69 | 20 | 36 | 0.74 | 2405 | 2182 | 0,099307247 | 0,097485423 | Northern Europe | Baltic States |
| Lithuania | 499 | 1 | 0 | 0 | 66,45 | 42.68 | 76.81 | 0.948 | 61 | 4 | 667000 | 44.476 | 21.750 | 12.18 | 4 | 65.284 | 46.9 | 5.86 | 68 | 7 | 32 | 1.11 | 3935 | 1946 | 0,244157746 | 0,0000956059 | Northern Europe | Baltic States |
| Luxembourg | 217 | 1 | 2 | 20 | 85,51 | 246.84 | 77.65 | 0.756 | 2 | 1 | 0 | 25.537 | 88.190 | 11.73 | 3 | 2.595 | 51.6 | 12.79 | 92 | 63 | 83 | 1.04 | 4941 | 1674 | -0,088683628 | 0,432317257 | Western Europe | Germanic Europe |
| Malta | 472 | 1 | 0 | 0 | 17,36 | 1.649 | 75.53 | 0.968 | 1 | 1 | 2973159 | 7.141 | 31.160 | 15.73 | 0 | 316 | 27.3 | 5.68 | 95 | 25 | 87 | 0.67 | 2161 | 2004 | 0,38720125 | 0,0000964403 | Southern Europe | Latin Europe |
| Moldova | 309 | 1 | 0 | 0 | 13,68 | 90.44 | 78.63 | 0.069 | 7 | 1 | 818 | 7.177 | 5.230 | 13.47 | 2 | 33.846 | 69 | 1.87 | 94 | 50 | 50 | 0.23 | 781 | 409 | 0,132063091 | 0,525358438 | Eastern Europe | Latin Europe |
| Netherlands | 3049 | 37 | 30 | 51 | 1013 | 520.73 | 79.42 | 6.25 | 29 | 5 | 15781756 | 623.369 | 55.180 | 12.13 | 2 | 37.377 | 53.8 | 5.02 | 93 | 75 | 73 | 2.31 | 6074 | 2367 | 0,441556662 | 0 | Western Europe | Germanic Europe |
| Norway | 2104 | 44 | 0 | 67 | 482,2 | 8.64 | 82.00 | 1.44 | 95 | 11 | 881238 | 99.253 | 83.190 | 32.06 | 3 | 385207 | 2.7 | 5.78 | 84 | 82 | 40 | 1.94 | 7228 | 3650 | 0,360873848 | 0 | Northern Europe | Nordic Countries |
| Poland | 1598 | 2 | 0 | 0 | 679,4 | 122.51 | 81.80 | 9.72 | 126 | 12 | 3187943 | 335.451 | 16.850 | 10.88 | 7 | 311.929 | 47.7 | 4.71 | 60 | 40 | 49 | 1.44 | 3534 | 2998 | 0,250374615 | 0,000000357628 | Eastern Europe | Slavic Europe |
| Portugal | 1208 | 10 | 2 | 2 | 253,7 | 112.7 | 80.02 | 6.35 | 64 | 8 | 3276794 | 99.337 | 23.880 | 9.74 | 1 | 89.103 | 43.3 | 4.40 | 67 | 44 | 42 | 1.68 | 5473 | 6306 | 0,428105742 | 0 | Southern Europe | Latin Europe |
| Romania | 633 | 2 | 0 | 0 | 284,1 | 81.08 | 77.46 | 5.02 | 45 | 16 | 631964 | 116.402 | 14.200 | 11.86 | 5 | 238.398 | 56.8 | 3.4 | 54 | 40 | 28 | 0.47 | 985 | 4241 | 0,316692859 | 0,000000119209 | Eastern Europe | Latin Europe |
| Serbia | 276 | 1 | 0 | 0 | 63,08 | 82.56 | 77.34 | 0.871 | 26 | 3 | 0 | 33.793 | 8.460 | 15.14 | 8 | 88499 | 41.4 | 2.75 | 57 | 30 | 91 | 0.99 | 2207 | 4704 | 0,191052899 | 0,109247684 | Southern Europe | Slavic Europe |
| Slovakia | 612 | 2 | 0 | 0 | 116,5 | 113.3 | 79.12 | 5.63 | 35 | 5 | 0 | 105.142 | 20.640 | 10.35 | 5 | 49.035 | 38.6 | 4.41 | 54 | 23 | 49 | 0.92 | 3211 | 4186 | 0,273378938 | 0,000000476837 | Eastern Europe | Slavic Europe |
| Slovenia | 924 | 2 | 0 | 0 | 61,75 | 103.49 | 81.91 | 1.83 | 16 | 3 | 1017788 | 49.067 | 28.280 | 13.16 | 4 | 20.273 | 30.3 | 4.9 | 56 | 49 | 39 | 2.13 | 5223 | 3795 | 0,239383221 | 0,00696516 | Southern Europe | Slavic Europe |
| Spain | 1859 | 161 | 7 | 246 | 1427 | 94.73 | 80.43 | 31.18 | 135 | 34 | 17712459 | 426.060 | 29.690 | 11.03 | 3 | 498.502 | 52.5 | 4.03 | 81 | 35 | 95 | 1.43 | 3252 | 6674 | 0,589520633 | 0 | Southern Europe | Latin Europe |
| Sweden | 2213 | 23 | 0 | 91 | 635,7 | 19.81 | 85.98 | 2.99 | 231 | 13 | 1635394 | 187.320 | 60.210 | 11.11 | 2 | 447.424 | 7.4 | 6.08 | 88 | 65 | 34 | 3.42 | 8131 | 2813 | 0,3242383 | 0 | Northern Europe | Nordic Countries |
| Switzerland | 773 | 6 | 6 | 1 | 800,6 | 210.49 | 80.54 | 4.39 | 63 | 6 | 114075 | 323.356 | 88.910 | 10.67 | 9 | 41285 | 37.9 | 4.47 | 74 | 94 | 58 | 3.36 | 6023 | 3203 | 0,402632147 | 0 | Western Europe | Germanic Europe |
| Ukraine | 1299 | 4 | 0 | 0 | 200,1 | 75.63 | 76.52 | 3.97 | 215 | 13 | 1022376 | 69.963 | 4.130 | 17.24 | 7 | 603628 | 71.3 | 2.66 | 70 | 42 | 33 | 0.29 | 587 | 5857 | 0,361062109 | 0,000000119209 | Eastern Europe | Slavic Europe |
| UnitedKingdom | 3744 | 78 | 21 | 406 | 3131 | 277.05 | 81.65 | 6.29 | 460 | 21 | 9844540 | 688.237 | 44.790 | 6.5 | 1 | 243610 | 71.2 | 4.20 | 84 | 72 | 57 | 2.91 | 4491 | 4557 | 0,554476202 | 0 | Western Europe | Germanic Europe |

**Supplement 2.** Detailed contribution of

|  | **Class** | | | | | | | | | | | | | | **Phyla** | | | | | | | | | | | |
| --- | --- | --- | --- | --- | --- | --- | --- | --- | --- | --- | --- | --- | --- | --- | --- | --- | --- | --- | --- | --- | --- | --- | --- | --- | --- | --- |
| **Country** | Actinopterygii | Agaricomycetes | Arachnida | Aves | Basidiomycota | Gastropoda | Insecta | Liliopsida | Magnoliopsida | Malacostraca | Mammalia | Pinopsida | Pucciniomycetes | Other_Class | Annelida | Arthropoda | Ascomycota | Basidiomycota | Chlorophyta | Chordata | Mollusca | Myzozoa | Nematoda | Rhodophyta | Tracheophyta | Other_Phylum |
| Albania | 26 | 0 | 0 | 0 | 0 | 0 | 17 | 25 | 129 | 0 | 8 | 0 | 0 | 23 | 0 | 20 | 0 | 0 | 3 | 37 | 0 | 0 | 0 | 4 | 159 | 5 |
| Austria | 0 | 0 | 0 | 0 | 55 | 58 | 399 | 155 | 642 | 0 | 0 | 0 | 0 | 299 | 0 | 496 | 0 | 55 | 0 | 69 | 61 | 0 | 0 | 0 | 804 | 68 |
| Belarus | 28 | 0 | 0 | 0 | 0 | 0 | 32 | 101 | 466 | 0 | 15 | 0 | 0 | 47 | 6 | 48 | 0 | 0 | 0 | 51 | 6 | 0 | 0 | 0 | 574 | 4 |
| Belgium | 0 | 0 | 0 | 242 | 36 | 0 | 147 | 377 | 1488 | 0 | 0 | 0 | 0 | 424 | 0 | 260 | 0 | 36 | 0 | 341 | 55 | 0 | 0 | 0 | 1914 | 72 |
| Bosniaand Herzegovina | 21 | 0 | 0 | 0 | 0 | 0 | 8 | 48 | 224 | 0 | 8 | 0 | 0 | 13 | 1 | 8 | 3 | 0 | 0 | 33 | 0 | 0 | 0 | 0 | 275 | 2 |
| Bulgaria | 37 | 0 | 0 | 0 | 0 | 0 | 171 | 87 | 647 | 0 | 0 | 0 | 0 | 135 | 0 | 188 | 0 | 0 | 0 | 57 | 0 | 27 | 0 | 0 | 740 | 65 |
| Croatia | 44 | 0 | 0 | 0 | 0 | 0 | 125 | 89 | 516 | 0 | 0 | 0 | 0 | 146 | 0 | 150 | 0 | 0 | 0 | 69 | 21 | 0 | 0 | 0 | 629 | 51 |
| Cyprus | 67 | 0 | 0 | 0 | 0 | 0 | 135 | 34 | 191 | 0 | 0 | 0 | 0 | 184 | 20 | 169 | 0 | 0 | 0 | 99 | 40 | 0 | 0 | 0 | 228 | 55 |
| Czechia | 0 | 0 | 53 | 0 | 0 | 0 | 424 | 140 | 879 | 0 | 0 | 0 | 0 | 274 | 0 | 501 | 0 | 0 | 0 | 81 | 37 | 0 | 0 | 0 | 1030 | 121 |
| Denmark | 0 | 0 | 0 | 0 | 0 | 0 | 435 | 283 | 1601 | 0 | 0 | 51 | 0 | 323 | 22 | 515 | 0 | 0 | 0 | 94 | 34 | 0 | 0 | 0 | 1941 | 87 |
| Estonia | 0 | 0 | 0 | 0 | 0 | 0 | 135 | 75 | 590 | 0 | 0 | 16 | 0 | 123 | 0 | 165 | 0 | 0 | 0 | 35 | 12 | 0 | 12 | 0 | 685 | 30 |
| Finland | 24 | 0 | 0 | 0 | 7 | 0 | 50 | 27 | 178 | 0 | 0 | 0 | 0 | 81 | 0 | 68 | 0 | 7 | 0 | 48 | 0 | 0 | 0 | 0 | 211 | 26 |
| France | 0 | 0 | 0 | 0 | 74 | 0 | 842 | 355 | 1603 | 93 | 0 | 0 | 0 | 795 | 0 | 1023 | 0 | 74 | 0 | 220 | 96 | 0 | 0 | 0 | 1997 | 278 |
| Germany | 0 | 90 | 0 | 0 | 91 | 0 | 483 | 185 | 987 | 0 | 0 | 0 | 0 | 674 | 0 | 667 | 0 | 91 | 0 | 149 | 91 | 0 | 0 | 0 | 1198 | 223 |
| Greece | 90 | 0 | 0 | 0 | 0 | 0 | 83 | 99 | 315 | 0 | 0 | 0 | 0 | 322 | 43 | 140 | 0 | 0 | 0 | 137 | 69 | 0 | 0 | 0 | 416 | 104 |
| Hungary | 25 | 0 | 0 | 0 | 6 | 0 | 78 | 41 | 148 | 60 | 0 | 0 | 0 | 60 | 0 | 143 | 0 | 6 | 0 | 44 | 13 | 0 | 0 | 0 | 195 | 11 |
| Iceland | 0 | 9 | 0 | 0 | 9 | 0 | 42 | 17 | 83 | 0 | 0 | 0 | 0 | 50 | 0 | 53 | 0 | 9 | 0 | 19 | 6 | 0 | 0 | 0 | 103 | 11 |
| Ireland | 0 | 0 | 0 | 0 | 0 | 40 | 269 | 162 | 952 | 0 | 0 | 0 | 0 | 290 | 0 | 315 | 0 | 0 | 0 | 81 | 49 | 0 | 0 | 0 | 1167 | 101 |
| Italy | 98 | 0 | 0 | 0 | 0 | 0 | 654 | 249 | 876 | 0 | 0 | 0 | 0 | 618 | 68 | 772 | 0 | 0 | 0 | 239 | 68 | 0 | 0 | 0 | 1164 | 184 |
| Latvia | 26 | 0 | 0 | 0 | 0 | 0 | 87 | 43 | 466 | 0 | 0 | 0 | 0 | 107 | 0 | 107 | 0 | 0 | 0 | 62 | 22 | 0 | 0 | 0 | 518 | 20 |
| Lithuania | 21 | 0 | 0 | 0 | 16 | 0 | 58 | 36 | 297 | 0 | 0 | 0 | 0 | 87 | 0 | 79 | 5 | 16 | 0 | 43 | 0 | 0 | 0 | 0 | 339 | 17 |
| Luxembourg | 9 | 0 | 0 | 0 | 0 | 0 | 36 | 18 | 112 | 0 | 0 | 0 | 0 | 42 | 0 | 48 | 1 | 0 | 0 | 26 | 3 | 0 | 0 | 0 | 135 | 4 |
| Malta | 37 | 0 | 0 | 0 | 0 | 0 | 66 | 57 | 202 | 0 | 0 | 0 | 0 | 110 | 0 | 85 | 0 | 0 | 0 | 58 | 24 | 0 | 0 | 0 | 267 | 38 |
| Netherlands | 0 | 0 | 76 | 0 | 52 | 0 | 359 | 399 | 1616 | 0 | 0 | 0 | 0 | 599 | 0 | 548 | 0 | 52 | 0 | 139 | 50 | 0 | 0 | 0 | 2090 | 170 |
| Norway | 0 | 60 | 0 | 0 | 66 | 0 | 240 | 178 | 1224 | 0 | 0 | 0 | 0 | 402 | 0 | 302 | 47 | 66 | 0 | 97 | 0 | 0 | 0 | 0 | 1455 | 137 |
| Poland | 0 | 0 | 0 | 59 | 77 | 0 | 341 | 70 | 629 | 0 | 0 | 0 | 0 | 499 | 0 | 449 | 0 | 77 | 0 | 143 | 0 | 0 | 0 | 0 | 713 | 216 |
| Portugal | 0 | 0 | 0 | 75 | 0 | 0 | 168 | 109 | 524 | 0 | 0 | 0 | 0 | 332 | 0 | 210 | 0 | 0 | 0 | 154 | 33 | 0 | 0 | 44 | 674 | 93 |
| Romania | 41 | 0 | 0 | 0 | 0 | 0 | 103 | 40 | 282 | 0 | 0 | 0 | 0 | 167 | 0 | 149 | 0 | 0 | 0 | 63 | 20 | 0 | 0 | 0 | 332 | 69 |
| Serbia | 25 | 0 | 0 | 0 | 0 | 0 | 39 | 25 | 142 | 0 | 0 | 0 | 0 | 45 | 0 | 46 | 6 | 0 | 0 | 36 | 9 | 0 | 0 | 0 | 170 | 9 |
| Slovakia | 35 | 0 | 0 | 0 | 6 | 0 | 51 | 61 | 413 | 0 | 15 | 0 | 0 | 37 | 0 | 66 | 3 | 6 | 0 | 56 | 0 | 0 | 0 | 0 | 478 | 3 |
| Slovenia | 0 | 0 | 0 | 0 | 117 | 0 | 195 | 56 | 293 | 0 | 0 | 0 | 60 | 320 | 0 | 208 | 103 | 117 | 0 | 89 | 0 | 0 | 0 | 0 | 358 | 49 |
| Spain | 0 | 0 | 0 | 202 | 0 | 0 | 247 | 196 | 767 | 0 | 0 | 0 | 0 | 447 | 0 | 328 | 0 | 0 | 0 | 330 | 54 | 0 | 0 | 0 | 990 | 157 |
| Sweden | 0 | 0 | 0 | 48 | 0 | 0 | 308 | 165 | 1301 | 0 | 0 | 0 | 0 | 390 | 0 | 367 | 0 | 0 | 0 | 140 | 48 | 0 | 0 | 0 | 1514 | 143 |
| Switzerland | 0 | 46 | 0 | 0 | 71 | 0 | 96 | 66 | 342 | 0 | 0 | 0 | 0 | 223 | 0 | 127 | 53 | 71 | 0 | 74 | 0 | 0 | 0 | 0 | 411 | 37 |
| Ukraine | 68 | 0 | 0 | 0 | 0 | 56 | 0 | 109 | 728 | 0 | 0 | 0 | 0 | 338 | 0 | 127 | 0 | 0 | 0 | 112 | 76 | 0 | 0 | 0 | 847 | 137 |
| UnitedKingdom | 0 | 0 | 0 | 504 | 0 | 0 | 627 | 325 | 1619 | 0 | 0 | 0 | 0 | 668 | 0 | 767 | 0 | 0 | 0 | 638 | 90 | 0 | 0 | 0 | 2006 | 242 |

**Supplement 3.** Network analysis based on the similarity in percentage composition of established non-native species classes at the country level. Nodes are scaled according to the number of established non-native species and coloured by geographic region.


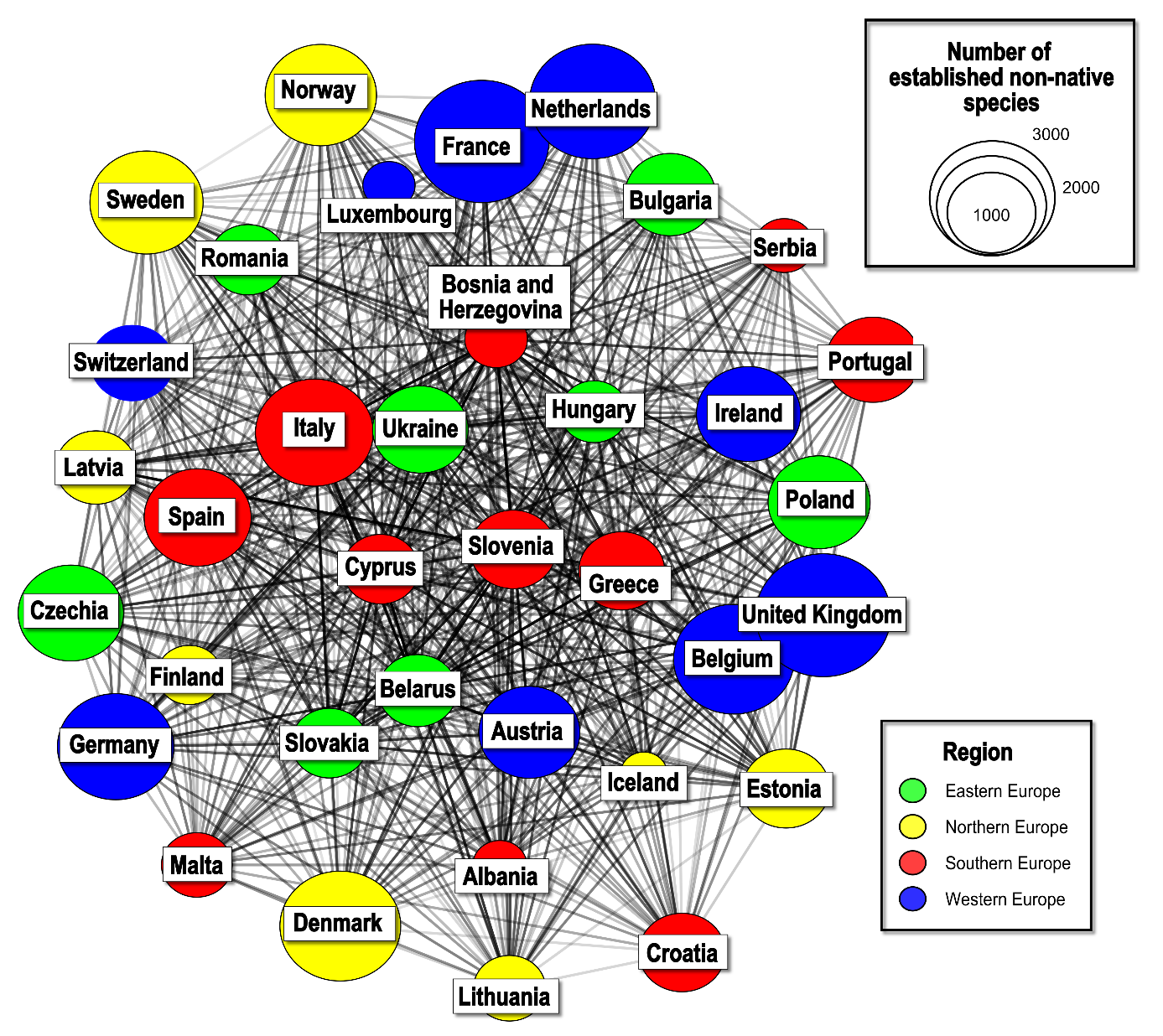


**Supplement 4.** Generalized linear model results for the number of established non-native species (model m1).

| Coefficients: | | | | |
| --- | --- | --- | --- | --- |
| model m1 | Estimate | Std.Error | t-value | Pr(>\|t\|) |
| (Intercept) | -7158.4601 | 2434.4694 | -2.940 | 0.006257 |
| n_timeseries | 4.6236 | 1.1014 | 4.198 | 0.000221 |
| Pop_dens | 4.1923 | 0.8447 | 4.963 | 2.59e-05 |
| Sust_development | 93.2777 | 28.8415 | 3.234 | 0.002965 |
| tourists_m | 27.3919 | 9.8646 | 2.777 | 0.009369 |
| Country_Growth | 40.8576 | 20.2185 | 2.021 | 0.052304 |
| Trompenaar_7 | -8.5809 | 4.4773 | -1.917 | 0.064868 |
| (Dispersion parameter for gaussian family taken to be 238500.7) | | | | |
| Null.deviance: 36104265 on 36 degrees of freedom | | | | |
| Residual.deviance: 7155020 on 30 degrees of freedom | | | | |
| AIC: 571.38 | | | | |

**Supplement 5.** Generalized linear model results for the trend in the reporting of established non-native species (model m2).

| Coefficients: | | | | |
| --- | --- | --- | --- | --- |
| model m2 | Estimate | Std.Error | t-value | Pr(>\|t\|) |
| (Intercept) | 2.013e-01 | 8.969e-02 | 2.244 | 0.03209 |
| GDP2021_b | 8.030e-05 | 2.279e-05 | 3.523 | 0.00135 |
| n_border_countries | -1.754e-02 | 8.246e-03 | -2.127 | 0.04150 |
| Ecological_footprint_hectat_pp | -2.493e-02 | 1.150e-02 | -2.167 | 0.03803 |
| Researcher_pp | 1.978e-05 | 1.074e-05 | 1.842 | 0.07501 |
| Nat_Biodiv | 4.851e-05 | 1.369e-05 | 3.543 | 0.00128 |
| (Dispersion parameter for gaussian family taken to be 0.01333562) | | | | |
| Null deviance: 1.1669 on 36 degrees of freedom | | | | |
| Residual deviance: 0.4134 on 31 degrees of freedom | | | | |
| AIC: -47.286 | | | | |

**Supplement 6.** Trajectories of significant Predictors for the number of established non-native species according to the applied generalised linear model.


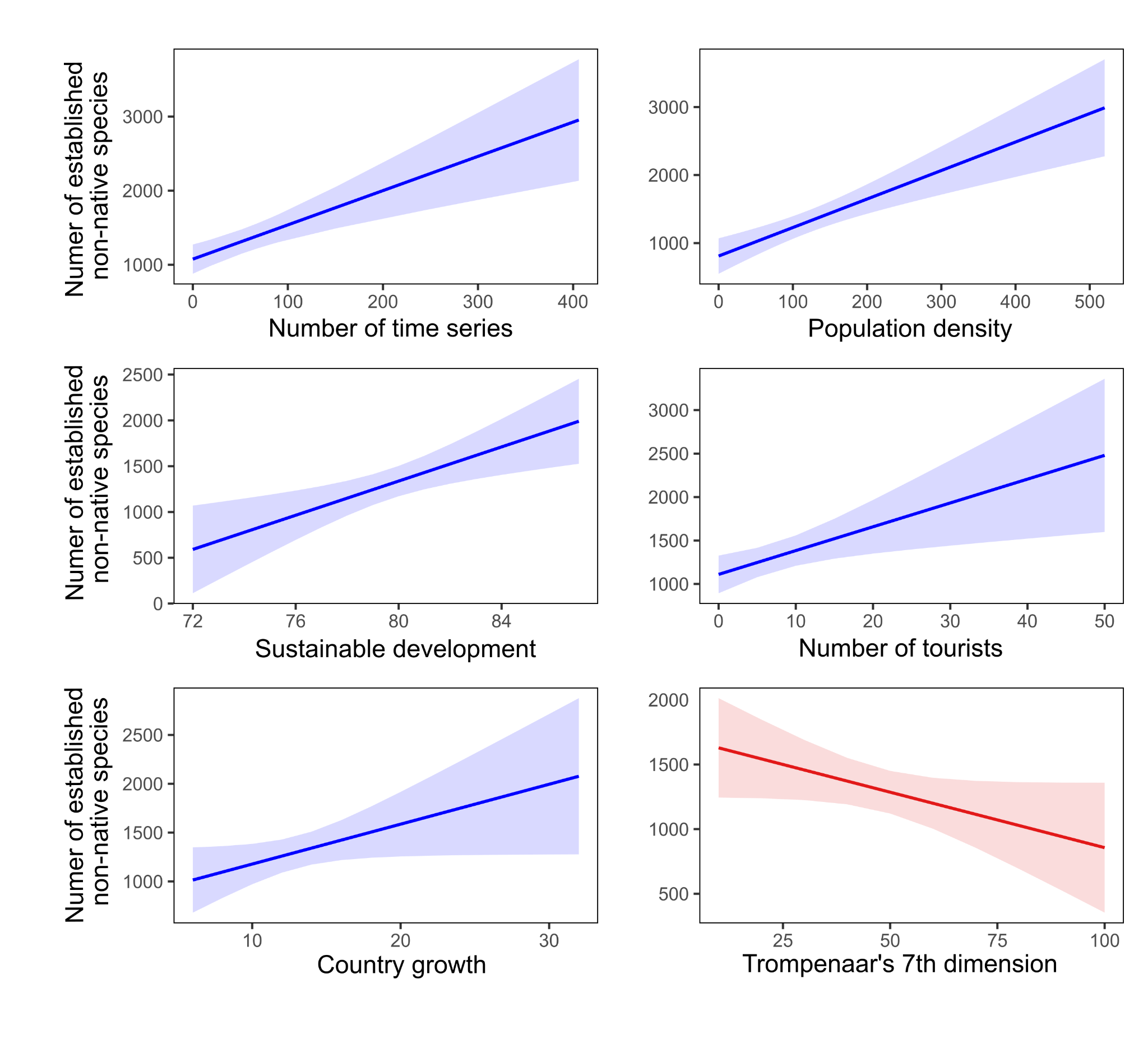


**Supplement 7.** Trajectories of sign. Predictors for the trend in the reporting of established non-native species according to the applied generalised linear model.


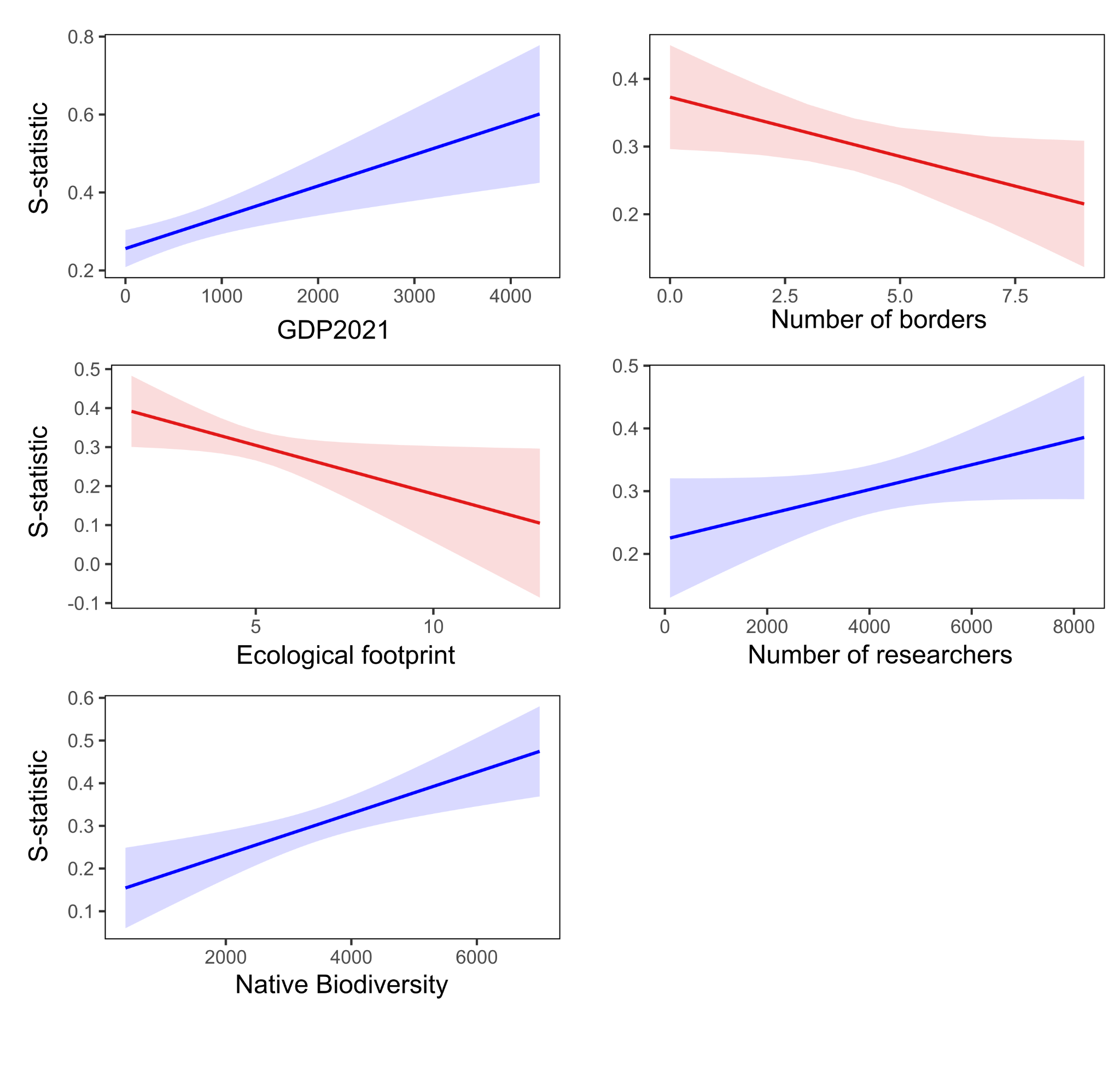

Supplement: Supplementary file 1 — Supplementary Material 1 [file 41598_2025_96133_MOESM1_ESM.docx]
